# Injuries in male and female semi-professional football (soccer) players in Nigeria: prospective study of a National Tournament

**DOI:** 10.1186/s13104-017-2451-x

**Published:** 2017-03-21

**Authors:** Oluwatoyosi Babatunde Alex Owoeye, Ayoola Ibifubara Aiyegbusi, Oluwaseun Akinleye Fapojuwo, Oluwaseun Abdulganiyu Badru, Anike Rasheedat Babalola

**Affiliations:** 1 Orthopaedic and Sports Physiotherapy Unit, Department of Physiotherapy, College of Medicine, University of Lagos, Idi-Araba, Yaba, Lagos State Nigeria; 2 Faculty of Kinesiology, University of Calgary, Calgary, Alberta Canada

**Keywords:** Epidemiology, Soccer, Injury prevention, Africa

## Abstract

**Background:**

Research on the epidemiology of football injuries in Africa is very sparse despite its importance for injury prevention planning in a continent with limited sports medicine resources. The vast majority of studies available in literature were conducted in Europe and only a very few studies have prospectively reported the pattern of football injury in Africa. The purpose of this study was to evaluate the incidence and pattern of injuries in a cohort of male and female semi-professional football players in Nigeria.

**Methods:**

A prospective cohort design was conducted, in which a total of 756 players with an age range of 18–32 years (356 males and 300 females) from 22 different teams (12 male and 10 female teams), were prospectively followed in a National Football Tournament. Physiotherapists recorded team exposure and injuries. Injuries were documented using the consensus protocol for data collection in studies relating to football injury surveillance.

**Results:**

An overall incidence of 113.4 injuries/1000 h (95% CI 93.7–136.0) equivalent to 3.7 injuries/match and time-loss incidence of 15.6 injuries/1000 h were recorded for male players and 65.9 injuries/1000 h (95% CI 48.9–86.8) equivalent to 2.2 injuries/match and time-loss incidence of 7.9 injuries/1000 h were recorded for female players. Male players had a significantly higher risk of injuries [IRR = 1.72 (95% CI 1.23–2.45)]. Injuries mostly affected the lower extremity for both genders (n = 81, 70% and n = 31, 62% for males and females respectively). Lower leg contusion (n = 22, 19%) and knee sprain (n = 9, 18%) were the most common specific injury types for male and female players respectively. Most of the injuries were as a result of contact with another player (n = 102, 88%—males; n = 48, 96%—females). Time-loss injuries were mostly estimated as minimal (n = 11, 69%) for male players and severe (n = 4, 66%) for female players.

**Conclusion:**

The overall incidence of injuries among Nigerian semi-professional football players is high but most of the injuries do not result in time-loss. Pattern of injuries is mostly consistent with previous studies. More prospective studies are needed to establish injury prevention initiatives among African players.

## Background

Nigeria has the highest number of male and female footballers (over 6.5 million players) in all categories of participation in Africa [1]. The importance of football in a developing country such as Nigeria cannot be overemphasized; football serves as a tool for personal and socioeconomic development. It has the potential to contribute to both personal and social development [2]. Furthermore, participation in football is an effective way for individuals to increase their level of physical activity and fitness. However, participation in a vigorous sport such as football at the competitive level exposes players to injuries that may blight their careers. The tragedy of a player sustaining a time-loss injury in a developing country such as Nigeria is unimaginable as medical care is very limited [3]. This indicates the need for effective strategies for prevention of injuries. Unfortunately, studies that may help understand the pattern of football injuries in order to effectively direct countermeasures for prevention are sparse in Nigeria and Africa at large.

Research on the epidemiology of football injuries in Africa is very sparse despite its importance for injury prevention planning in a continent with limited sports medicine resources. Numerous studies have been conducted to determine the type, site and severity of injuries sustained by football players, however, the vast majority of studies were conducted in Europe [4–18] and only a few studies have reported the pattern of football injury in Africa [19–28]. Few studies have so far prospectively reported the incidence and pattern of football injuries in African players [23–27]. These studies revealed match injuries to range from 186 to 289 injuries per 1000 match hours (equivalent to 6–10 injuries per match for all injuries) and 20–55 injuries per 1000 match hours for time-loss injuries for male players [24–26]. Only one study was found to prospectively document the incidence of injuries among female players in Africa using an established reporting system [27]. At present studies on the incidence of injuries in Africa remains sparse and there are no known prospective studies on African and Nigerian male and female semi-professional players.

An understanding of the incidence and characteristics of injuries sustained by players would be essential for the design of injury prevention programmes for this group of players. This study therefore prospectively evaluated injuries in a cohort of male and female Nigerian football players during the 18th National Sports Festival held in Lagos, Nigeria.

## Methods

### Study design, participants and setting

A prospective cohort design was adopted for this study. A total of 756 players with an age range of 18–32 years (356 males and 300 females) from 22 different teams (12 male and 10 female teams), registered for the 18th National Sports Festival Football Tournament participated in the study. The tournament took place in Lagos, Nigeria in November 2012. Tournament matches were played at three different locations in Lagos, Nigeria-the Teslim Balogun Stadium, Surulere, Yaba College of Technology Stadium, Yaba and the Agege Stadium, Agege, Lagos.

The National Sports Festival is a biennial event comprising several sports for young and upcoming athletes in Nigeria involving athletes from all states across the country. The teams who participated in the national finals represented states who qualified through playoffs conducted across the 6 geo-political zones of the country.

### Definition of injury and procedures for data collection

For the purpose of this study, injury was defined as any physical complaint during a match, which necessitated medical attention from the physiotherapist, regardless of consequences with respect to absence from the remaining matches; in accordance with the consensus statement on injury definitions for football injury surveillance [29]. However, time-loss from football play was estimated for each injury recorded. Physical complaints such as illnesses and diseases were not recorded and previous injuries sustained prior to camping were not recorded in this study. Injured players were followed up after every match to ensure complete documentation and full diagnosis and characteristics of injuries.

Injuries and exposure time were recorded by three physiotherapists officially assigned to tournament venues. Before the start of the tournament, the venue physiotherapists were instructed in detail how to complete the injury report form. They were provided with a study manual explaining definitions and instructions on data collection procedures. Three of the researchers (one per venue) supervised venue physiotherapists and were actively involved in follow-up assessments of injured players after each match. Injuries were documented using the injury report forms accredited by experts for injury surveillance studies relating to football [29].

Ethical and official approval to conduct this study was sought and obtained from Health Research and Ethics Committee of the Lagos University Teaching Hospital and the Local Organising Committee of the National Sports Festival respectively. The team coaches, players, and match venue physiotherapists were duly informed about the study and their consent was obtained prior to the football tournament.

### Data analyses

The incidence of injury was expressed as number of injuries per match and number of injuries per 1000 match-hours with 95% confidence intervals (CI) using Poisson model to estimate incidence rate ratio with 95% CI. The amount of hours of match play for each team was calculated as follows: 11 players × 1.5 h × number of matches, from which overall exposure time was derived. Descriptive statistics of frequency and percentage were used to summarize the distribution of injuries by characteristics and severity in accordance with the recommendations by Fuller et al. [29]. Data analysis was conducted using STATA (version 14.1, Collage Station, USA). Statistical significance was accepted at α = 0.05 level.

## Results

### Injury incidence

The incidence of injuries (all injuries and time-loss injuries) among players is presented in Table 1. A total of 116 injuries were recorded in 31 matches for male players, resulting in an overall incidence of 113.4 injuries/1000 match-hours (95% CI 93.7–136.0) equivalent to 3.7 injuries/match (95% CI 3.1–4.5). Sixteen (13.8%) injuries were expected to result in absence from play (time-loss). For the female players, a total of 50 injuries were recorded in 23 matches resulting in 65.9 injuries/1000 match-hours (95% CI 48.9 86.8) equivalent to 2.2 injuries/match (95% CI 1.6–2.9). Overall, male players had a significantly higher risk of injuries [IRR = 1.72 (95% CI 1.23–2.45)].

**Table 1:** Incidence of injuries in semi-professional football players in Nigeria

Variable	Males	Females
All injuries		
No of injuries	116	50
Match exposure (hours)	1023	759
Injuries per match (95% CI)	3.7 (3.1–4.5)	2.2 (1.6–2.9)
Injuries per 1000 h (95% CI)	113.4 (93.7–136.0)	65.9 (48.9–86.8)
IRR (95% CI)	1.72 (1.23–2.45)*	Reference
Time-loss injuries		
No of injuries	16	6
Injuries per match (95% CI)	0.5 (0.3–0.8)	0.3 (0.1–0.6)
Injuries per 1000 h (95% CI)	15.6 (8.9–25.4)	7.9 (2.9–17.2)
IRR (95% CI)	1.98 (0.74–6.17)	Reference

*IRR* incidence rate ratio

* Significant at p < 0.01

### Characteristics and severity

In male players, most of the injuries recorded affected the lower extremity (n = 81, 70%). Contusion injuries to the lower leg (n = 22, 19%) and ankle sprain (n = 15, 13%) were the most common specific injury types. Time-loss injuries were mostly reported to affect the head/neck (n = 4, 25%) and ankle (n = 4, 25%). Furthermore, ankle sprain (n = 3, 19%), knee sprain (n = 3, 19%) and concussion with loss of consciousness (n = 2, 13%) were specifically the most frequent time-loss injuries (Table 2). Most of the injuries were as a result of contact with another player (n = 102, 88%).

**Table 2:** Location and diagnosis of injuries

Location and diagnosis	Males All n (%)	Time-loss n (%)	Females All n (%)	Time-loss n (%)
Head/neck	20 (17)	4 (25)	9 (18)	1 (16.6)
Concussion	3	2	5	1
Contusion	11	1	4	0
Laceration/abrasion	6	1	–	0
Upper limb	7 (6)	0 (0)	6 (12)	0 (0)
Contusion	6	0	–	0
Sprain	1	0	3	0
Strain	–	0	2	0
Dislocation	–	0	1	0
Trunk	8 (7)	1 (6)	4 (8)	0 (0)
Contusion	8	1	2	0
Sprain	–	0	1	0
Strain	–	0	1	0
Hip/groin	5 (4)	1 (6)	3 (6)	1 (16.6)
Contusion	3	0	–	0
Sprain	–	0	1	0
Strain	2	1	2	1
Thigh	7 (6)	1 (6)	4 (8)	1 (16.6)
Strain	2	1	2	1
Contusion	4	0	2	0
Muscle cramp	1	0	–	0
Knee	18 (16)	3 (19)	10 (20)	1 (16.6)
Sprain	10	3	9	1
Contusion	8	0	1	0
Lower leg	29 (25)	2 (13)	3 (6)	0 (0)
Contusion	22	0	–	0
Muscle cramp	5	1	–	0
Strain	2	1	3	0
Ankle	21 (18)	4 (25)	5 (10)	1 (16.6)
Sprain	15	3	4	1
Contusion	5	0	–	0
Strain	–	0	1	0
Subluxation	1	1	–	0
Foot/toe	1 (1)	0 (0)	6 (12)	1 (16.6)
Contusion	1	0	5	0
Sprain	–	0	1	1
Total	*116*	*16*	*50*	*6*

In female players, injuries mostly affected the lower extremity (n = 31, 62%). Knee sprain (n = 9, 18%) was the most common specific injury type. Time-loss injuries were reported to evenly affect most body parts, but were mostly sprain injuries (3 of 6 injuries; 50%) (Table 2). Almost all the injuries were caused by player contact (n = 48, 96%).

### Injury severity

In most cases, players were able to continue playing in the male (100 of 116, 86%) and female tournaments (44 of 50, 88%). Injuries were mostly estimated as minimal (n = 11, 69%) for male players and severe (n = 4, 66%) for female players (Table 3).

**Table 3:** Severity of injuries in semi-professional football players in Nigeria

Characteristics	Males n (%)	Females n (%)	Total n (%)
Minimal (1–3 days)	11 (69)	0 (0)	11 (50)
Mild (4–7 days)	2 (12)	1 (17)	3 (14)
Moderate (8–28 days)	3 (19)	1 (17)	4 (18)
Severe (>28 days)	0 (0)	4 (66)	4 (18)
Total	16 (100)	6 (100)	22 (100)

Based on estimated days of absence from football participation

### Characteristics of time-loss injuries sustained by players

The characteristics of time-loss injuries as sustained by players are presented in Table 4. Time-loss injuries were mostly reported by midfielders (n = 12, 54%) affecting about two-thirds (n = 10, 62%) of male and a third (n = 2, 33%) of female midfield players. Time-loss injuries were only reported by 2 (33%) female goalkeepers while none were reported by the male goalkeepers. Time-loss injuries mostly occurred during the 2nd half of football matches for both genders (n = 16, 73%). Majority (n = 19, 86%) of the time-loss injuries were as a result of contact and were mostly sprains (n = 10, 45%).

**Table 4:** Characteristics of time-loss injuries sustained by players

Characteristics	Males n (%)	Females n (%)	Total n (%)
Player’s position			
Striker	2 (13)	1 (17)	3 (14)
Midfielder	10 (62)	2 (33)	12 (54)
Defender	4 (25)	1 (17)	5 (23)
Goalkeeper	0 (0)	2 (33)	2 (9)
Period of match			
1st Half	3 (19)	2 (33)	5 (23)
2nd Half	12 (75)	4 (67)	16 (73)
Extra time	1 (6)	0 (0)	1 (4)
Injury mechanism			
Contact	13 (81)	6 (100)	19 (86)
No contact	3 (19)	0 (0)	3 (14)
Injury type			
Sprain	6 (37)	4 (66)	10 (45)
Strain	3 (19)	1 (17)	4 (18)
Concussion	2 (13)	1 (17)	3 (13)
Contusion	2 (13)	0 (0)	2 (9)
Subluxation	1 (6)	0 (0)	1 (5)
Muscle cramp	1 (6)	0 (0)	1 (5)
Laceration	1 (6)	0 (0)	1 (5)

## Discussion

This prospective study assessed the incidence and characteristics of injuries in male and female amateur football players who participated at the 18th National Sport Festival that was held in November 2012, in Lagos State, Nigeria. This study is the first to document the incidence of injuries in a completely Nigerian football tournament. The consensus protocol for data collection in studies relating to football injury surveillance was used for this study in order to allow comparison of our results with other studies [29]. However, no prospective studies were found relating to adult female semi-professional football players in Nigeria or Africa, hence comparison in this regard was limited.

This study revealed a high overall injury incidence for both male and female football players. However, most (86 and 88% for male and female players respectively) of the injuries did not stop players from continuing in the tournament. An overall injury incidence of 113.4 and time-loss incidence of 15.6 injuries/1000 match-hours as recorded for male players is high in comparison with similar studies done outside Africa [4–18]. This result however corroborates previous studies reporting a high incidence of injury among male football players in Africa [24–26]. An overall incidence of 65.9 and time-loss incidence of 7.9 injuries/1000 h for female players is within the range recorded for female football players during top-level FIFA tournaments [30]. However, this result is lower than the injury incidence documented for female youth football players in Kenya [27].

Injury surveillance studies among male and female football players in the African continent seem unequivocal on high incidence of injuries among players and our study corroborates this [24–27]. The high incidence of injuries may be hinged on a few factors as discussed in previous studies [24, 25, 27]. One major factor attributable to increased incidence of injuries is the generally low level of skills and the physical pattern of play in African football. Based on the records of International football matches, skill levels in less developed settings such as Africa and Asia have been considered to be lower than those of their European counterparts [7]. Although the influence of the different skill levels on the incidences of injury remains a controversial issue, some researchers reported that lower skill levels were associated with more frequent occurrences of injuries [17]. While most reported injuries are not time-loss, the trend of high football injury incidence in the African region calls for urgent attention and appropriate intervention.

In the present study, majority of the injuries affected the lower extremities (70 and 61% for males and females respectively); mostly the lower leg, ankle and knee joints, which is in concordance with other reports [4–26, 30, 31]. The most common types of injuries were contusions, sprains, and strains; this is also in agreement with previous studies [4–26, 30, 31]. It is important to note that contusion injury accounted for three out of five injuries of all injuries; most of which did not result in time-loss. Furthermore, the ankle and knee joints recorded the highest proportion of time-loss injuries by male players. This is in congruence with previous studies on professional male football tournaments [6, 8, 12]. However, time-loss injuries were not specific to any body parts for female players.

In most cases, players were able to continue playing in the male and female tournaments. Hence, the focus on injury management during subsequent tournaments should be on the field treatment of non-severe injuries such as contusions. No severe injuries (time-loss over 4 weeks) were reported for male players but surprisingly, 66% of the few time-loss injuries reported for female players were estimated as severe injuries. This finding of high rate of severe time-loss injuries among female players is in contrast with a previous study carried out on young female Kenyan players in which no severe injuries were reported [27] and another study carried out on male youth football players in Nigeria in which 9.8% of the injuries were reported as severe in the control sample of a randomised controlled study [26]. More prospective studies are needed to fully understand whether this trend is a peculiar one in female football in Nigeria.

Due to the impact of time-loss injuries on football players, a separate evaluation was done to reveal specific characteristics of time-loss injuries sustained by players. Time-loss injuries were mostly reported by midfield players for both genders although tied with goalkeepers for female players. Report of which player position is more at risk for injuries varies with different studies and geographical locations. Comparable studies [24–27] in African players did not focus on this aspect of our study. Practically, midfielders’ role to succeed derive their abilities through slickly, quick and decisive skills coupled with effort to succeed in retrieval or defending of the ball. This suggests that the midfielders known for their multitasking role and high work rate at every match are exposed to injuries more than any player position. Furthermore, midfield roles require utmost concentration, close marking, agility and speed which increase the injury risk. Thus, it is important that midfielders in this population of players are given special attention for injury prevention and proper injury rehabilitation. In consistence with previous studies, players sustained more injuries in the second half of matches than in the first half [8–10].

The present study is limited in scope to semi-professional football players in Nigeria and it only presents acute injuries sustained during matches; hence the present study has its limitations. To fully understand the extent of the problem of acute and overuse injuries in Nigerian and African football players, there is a need for more prospective studies. Future studies should consider reporting both acute and overuse injuries using appropriate surveillance methodologies at the various levels of competition for both match and training injuries during tournaments and league seasons. Understanding the risk factors peculiar to injuries in Nigerian and African football is also valuable in planning prevention initiatives for players. The present study gives an insight to the pattern of match injuries among semi-professional players in an African population and generally adds to the scanty literature on the epidemiology of football injuries in Africa.

## Conclusion

The overall incidence of injuries among Nigerian semi-professional football players is high. Most of the injuries did not result in time-loss for both genders. Time-loss injuries were mostly minimal in male players but severe in female players. Pattern of injuries is mostly consistent with previous studies. More prospective studies are needed to fully understand the pattern of injuries and establish prevention strategies among African players.
